# Relationship of Cultivated Grain Amaranth Species and Wild Relative Accessions

**DOI:** 10.3390/genes12121849

**Published:** 2021-11-23

**Authors:** Ranjita Thapa, Matthew Edwards, Matthew W. Blair

**Affiliations:** 1 Department of Agricultural and Environmental Sciences, Tennessee State University, Nashville, TN 37209, USA; thaparanjita2@gmail.com (R.T.); mve521@aol.com (M.E.); 2 Plant Pathology and Plant-Microbe Biology Section, Cornell University, Geneva, NY 14456, USA

**Keywords:** cultivated and wild *Amaranthus*, competitive allele specific PCR, domestication processes, genetic diversity, weed-crop complexes and relatives

## Abstract

*Amaranthus* is a genus of C4 dicotyledonous herbaceous plants, and three New World species have been domesticated to produce grain crops with light colored seed which are classified as pseudo-cereals rich in protein and minerals. A core collection of grain amaranths and immediate precursor species has been established, representing the closest related species. The goal of this study was to evaluate the genetic diversity in that collection of cultivated and wild species, using competitive allele single nucleotide polymorphism markers. A secondary objective was to determine the relationships among the three cultivated species and non-domesticated *Amaranthus*, while a third objective was to evaluate the utility of the markers in detecting diversity in the 276 genotypes. The markers were found to be highly variable with an average polymorphism information content of 0.365. All markers were bi-allelic; and the major allele frequency ranged from 0.388 to 0.871. Population structure analysis of the cultigens revealed the presence of two sub populations. Phylogeny confirmed that the two Mesoamerican species, *Amaranthus cruentus* and *Amaranthus hypochondriacus,* were related and distant from the South American species *Amaranthus caudatus*, which in turn was very closely clustered with *Amaranthus quitensis,* even though this is considered a weedy relative. The first pair of species were likely to have inter-crossed, while the latter two likely exist in a wild-cultivated hybrid state. In conclusion, the results of this SNP study provided insights on amaranth cultivars and their relationship to wild species, the probable domestication events leading to the cultivars, and possible crop breeding or germplasm conservation strategies.

## 1. Introduction

Amaranths belong to the genus *Amaranthus* L., which is made up of approximately 70 species of C_4_ dicotyledonous herbaceous plants [1,2]. About 60 species are native to the Americas; while 10 others are from Asia, Africa, Australia and Europe [3]. The genus *Amaranthus* contains cultivated, weedy and wild species.

Cultivated amaranths are used for grain, vegetables, forages and ornamental plants, but food grain and leafy vegetables are the most ancient uses [2,4]. The first of these have been cultivated for more than 8000 years in the region of Mesoamerica and the Andes mountains [5]. The major species are *A. caudatus*, *A. cruentus* and *A. hypochondriacus*, and their corresponding parental wild species are thought to be *A. hybridus*, *A. quitensis* and *A. powelli*, respectively [2]. 

Grain amaranths are known to be highly nutritious [6,7,8,9] and have good nutraceutical properties [10,11,12,13]. Domestication of grain amaranths is thought to have occurred primarily in Mexico and South America, and is still important to indigenous communities in those regions [3]. 

These pseudocereal, grain amaranth species are also a subsistence food crop for people living in Eastern and Southern Africa [9,14], and a commercial crop for East and South Asia [2,15]. Grain amaranths have the advantage of being useful as boiled, popped, malted or fermented foods [9,16]. Given their importance for a large area and for multiple dietary needs, germplasm collections and molecular characterization of grain amaranths are important for developing new varieties. Knowledge of relative gene diversity among and within wild populations would be useful in plant breeding of amaranths and ex situ conservation strategies for this crop [4]. More specifically, genetic diversity assessment of amaranths helps in the identification of diverse parental combinations to use in breeding programs, which then helps plant breeders to create segregating progenies with a maximum genetic variability [17]. Well-characterized, phenotyped accessions such as those in core collections, facilitate introgression of desirable alleles from diverse germplasm into a commercial varieties for this crop as for other plants [18].

Different morphological traits and molecular markers have been used in genetic diversity analyses of grain amaranths e.g., [19,20]. Among morphological markers, growth characteristics have been used; however, these are influenced by environmental factors. Misidentification is common with morphology because there is a range of plasticity of descriptors for pigmentation, flower and leaf morphology among grain amaranth species. This phenotypic plasticity creates non-genetic variation within cultivated accessions [3,19]. Therefore, insufficient distinctive characters, and ecotype variability limit the use of morphological markers and reinforce the need for molecular markers for identification of species, cultivars, accessions and hybrids.

Several single locus molecular markers have been used for amaranth characterization instead of morphological ones. These range from protein-based ones to DNA-based markers, including isozymes [20,21,22], RFLPs [23], SSRs [1,24,25] and SNPs [26,27]. Multi-locus markers have included RAPDs [28,29,30] and AFLPs [31]. Some of these markers had drawbacks for amaranth genetics. For example, RAPD markers, being dominant, could not distinguish between homozygotes and heterozygotes, and were not always repeatable. RFLPs proved impractical as they required a large amount of DNA. AFLPs had a requirement of higher molecular weight DNA as well as dominant scoring, making them useful in populations of amaranths. Isozymes were never widespread in amaranths because of restricted number of suitable loci. Simple sequence repeat (SSR) markers are somewhat species specific to each amaranth group so cannot be used across relatives.

Single nucleotide polymorphism (SNP) markers have emerged as one of the most powerful and easy to use fingerprinting systems for crop genetic studies especially for the study of related accessions in population genetics [32]. SNPs are single base changes in the genetic codes at specified loci and are the most abundant type of sequence variation in higher plants [33]. SNPs have a low mutation rate, are bi-allelic and adaptable to high throughput genotyping at low cost. High-throughput SNP genotyping is carried out by various techniques. Genotyping by sequencing (GBS) for grain amaranths has been important because it has provided a greater number of markers than previous [34,35].

In this study, we decided to use competitive allele (KASP) technology for various reasons. KASP have flexibility in evaluating many genotypes at a time [26]. They also benefit from real-time data generation with no toxic dyes or gel substances. Perhaps most importantly, KASP are inexpensive per datapoint using low PCR volume requiring fewer reagents compared to other assays [36]. Single SNP markers are valuable as long-term breeding tools because they are technically repeatable and efficient for scaling up. A significant number of KASP markers are available for grain amaranth based on SNP loci found for *A. caudatus* accessions [26] and then mapped onto 16 linkage groups [27]. Over 400 KASP from those studies were developed primarily for evaluation of landraces of grain amaranths from the Andes, and 96 were used for a study of South American grain amaranths [37], which identified two groups among 178 Peruvian amaranth accessions belonging to *A. caudatus* and *A. hybridus* based on seed color (black, brown, and white-vitreous versus white-opaque). 

That study did not find distinct genotypic groups based on geographic origin. Interestingly, outcrossing was more common in the wild type seed: observed heterozygosity (H_o_) was 0.2612 in the vitreous-seeded subgroup while expected heterozygosity (H_e_) was 0.398 in the brown-seeded subgroup [37].

Our study aimed to test the KASP markers in a wider panel of genotypes including *A. cruentus* and *A. hypochondriacus.* For that, we selected the highest polymorphism information content (PIC) value markers from the previous SNP analyses to use in an assessment of the diverse collection of amaranths which included many accessions of all three cultivated grain amaranth species and their closest wild relatives. Our specific objectives were to evaluate the performance of the SNPs in detecting genetic diversity and relationships among a total of 276 *Amaranthus* genotypes and to determine the level of separation versus admixture in population structure as it is related to the species differences.

## 2. Materials and Methods

### 2.1. Plant Material

A total of 276 accessions of *Amaranthus* were selected representing highly diverse grain amaranths based on our previous morphological study [38]. These accessions belonged to 9 different species. Of these, 249 were from the core collection established by the USDA National Plant Germplasm System in Ames, Iowa (Supplementary Materials, Table S1); while 27 were from Seed Savers’ Exchange (SSE), United States cultivars (Supplementary Materials, Table S2). The plant introduction (PI) accessions had been collected from Asia, Africa, Europe, Central America, South America and North America and represented 35 countries around the world; while the cultivars were from SSE located in Decorah, Iowa. Despite the geographical diversity of collection sites, most of the genotypes were from just two countries: Mexico and Peru. In terms of species identification, genotypes from USDA represented a) cultivated grain amaranth species accessions including 120 accessions of *A. cruentus*, 44 accessions of *A. hypochondriacus* and 33 accessions of *A. caudatus*; and b) wild relative or weedy amaranth species accessions, including 26 of *A. hybridus*, 16 of *A. quitensis*, 6 of *A. powellii*, 2 of *A. retroflexus* and 1 of *A. palmeri*. In addition, 1 accession of *A. australis*, a wild swamp amaranth from Florida, was used in the study. In summary, all the grain amaranth accessions represented cultivated landraces or genotypes from farmers’ fields; while all the other species were wild or weed collected. In total, among the 276 accessions, 52 were weedy/non-grain types.

### 2.2. DNA Extraction of In Vitro Grown Seedlings

A unique method of DNA extraction was developed in this project for the following reason: grain, weedy or wild amaranth plants are slow growing in their initial seedling stages when sown in soil and do not produce large leaves adequate for DNA extraction until three weeks after planting under those conditions. Therefore, an in vitro plant culture method was developed to collect fresh leaf tissues for the extraction procedure.

The seeds of all 276 *Amaranthus* accessions were germinated under aseptic conditions in magenta boxes filled with 100 mL. M.S. media [39] with 2 g sucrose per box. Prior to seeding, 25 seeds from each accession were surface sterilized with a 30% *v/v* Chlorox: bleach (Sodium Hypochlorite, NaClO solution): double distilled (Millipore) water solution for 5 min followed by five rinses with autoclaved water.

The clean seeds were placed in the magenta boxes in a sterile laminar air flow hood. These boxes were sealed with parafilm and then placed in a Nor-Lake Sci. growth chamber (Nor-Lake Inc., Hudson, WI, USA), which was maintained at 16 hr. light photoperiod and 25 °C constant temperature. After two weeks the seedlings were harvested, and leaves removed from stem and hypocotyl tissue. Genomic DNA of each *Amaranthus* accession was extracted from the leaves using FASTDNA^®^ miniprep kits (MP Biomedical, Solon, OH, USA). 

The concentration and quality of the DNA samples was measured by NanoDrop 1000 UV-Vis Spectrophotometer (Thermo Fisher Scientific Inc., Waltham, MA, USA) and DNA samples were diluted with autoclaved ultrapure water to prepare working stocks of 10 ng/ μL for SNP genotyping.

### 2.3. SNP Marker Analysis

We used a total of 45 SNP markers previously designed for KASP assays and registered as highly polymorphic in both *A. caudatus* and *A*. *hybridus* by Maughan et al. (2011). The SNP markers represented loci that were evenly distributed across the 16 chromosomes of the amaranth genome. The oligo-nucleotide mixtures for these markers were ordered as KASP by design (KBD) genotyping assays from LGC Limited (Beverly, MA, USA). Each KBD consisted in three oligonucleotides surrounding the SNP locus and able to detect two states depending on the alternative nucleotides present there. The three oligonucleotides for each assay were dissolved in 10 mM Tris-HCl (pH = 8) to a 100 μM concentration, mixed together as a SNP assay mix (12 μL AS1-primer1, 12 μL AS2-primer2, 30 μL CP-common, in 46 μL Tris-HCl pH8) and 2 μL aliquots were distributed into individual wells of 96 well plates. Assay plates were frozen at −20 °C until use.

The PCR cycling was performed in 96- well skirted PCR plates, with a total reaction volume of 10 µl for each reaction containing 5µl genomic DNA (10 ng/µl). The PCR plate was sealed with an optically transparent plastic seal using a KUBE Sealer machine (LGC genomics, Sheffield, U.K.). Each SNP was genotyped in a total reaction volume of 4 μL in the following reaction mixture: 6 ng DNA, 22 mM MgCl_2_, 0.5 unit of *Taq* enzyme, 1 μL 4× reaction mix, and 2 μL pre-plated 1× assay mix. PCR amplification were carried out in an Eppendorf 100 machine (Eppendorf, Hauppauge, NY, USA) using the amplification conditions that were recommended by Maughan et al. (2011); whereby, thermal cycling consisted of a hot-start *Taq* polymerase activation step (94 °C for 15 min) followed by a sub sequent touchdown amplification protocol, which consisted of 10 cycles of 94 °C for 20 sec, 65 °C for 1 min (decreasing 0.8 °C per cycle), followed by 26 cycles of 94 °C for 20 sec and 57 °C for 1 min. The final temperature was reduced to 20 °C for 30 sec, and then the program was shut down with plates removed at room temperature soon thereafter. Subsequently, the reactions were fluorescently scanned within a day of PCR as described below.

### 2.4. SNP Calling and Data Analysis

After amplification, the 96 well plates were put in the exposure cabinet of a FLUOstar Omega fluorescence plate reader (BMG Labtech Inc., Cary, NC, USA) to read end-point fluorescent images using appropriate wavelengths for KASP assay dyes. Marker genotyping was visualized and interpreted using KlusterCaller software (LGC Ltd., London, UK). The results were translated into nucleotides observed at each SNP locus for a data matrix of allele calls that was down loaded to Excel and used to calculate polymorphism information content (PIC) first [40]. Followed by genetic diversity, allele number and major allele frequency assessment using POWER MARKER v. 3.25 [41]. PIC values were based on number of alleles and the frequency of alleles for each marker.

Following marker characterization, diversity evaluation of the cultivated grain amaranth accessions alone was done with population structure analysis in STRUCTURE v.2.3.3 software [42]. The program was run with no a priori genotype assignments, but with different numbers of sub-populations (K) ranging from 1 to 10 and with 100,000 burn ins and 200,000 Markov chain Monte Carlo (MCMC) iterations. Each K-value was performed using admixture models with five independent simulation runs. Average likelihood value, L(K) across all runs was calculated for each K-value. Evanno test [43] was used to determine the optimum K number. Genotypes were assigned to subpopulations based on the likelihood within each population [44]. An individual with a threshold value of more than 85% genome fraction was assigned to a population. 

Following this, an unweighted pair group method with arithmetic mean (UPGMA) dendrogram was drawn to display clustering of all the genotypes based on DARwin software (https://darwin.cirad.fr/, accessed on 17 November 2021) using default similarity indices. 

Phylogenetic analyses for the different species were performed with POPGENE32 software using Nei coefficient [45]. Supplementary Materials, Figure S1 shows the geographic distribution of the species with collection site data and the diverse altitude and latitudes from which the accessions were sampled. 

Genetic variation within and among species, and within and among the country of origin was identified for 249 accessions from USDA using an analysis of molecular variance (AMOVA) based on GenAlex v.6.51 software [46]. *A. palmeri* and *A. australis* had only one accession, and hence were removed from the AMOVA analysis. 

Pairwise estimates of the correlation of alleles among individuals within subpopulations (F_IS_), fixation index among subpopulations within the total population (F_ST_), and fixation index among individuals within the total population (F_IT_) were calculated.

## 3. Results

### 3.1. Characterization of SNP Markers

As this was a study of many genotypes, 45 of the best KASP markers from the AM series [26,27] were selected considering polymorphism and genome location [47]. All but one of the SNP markers amplified well and were detectable under the standard PCR and fluorescent detection conditions and techniques we used. Only one SNP marker (AM19583) did not amplify well with our PCR conditions and was not considered further in data analyses. For the 276 DNA samples × 44 SNP marker combinations that amplified, all were polymorphic. In addition, all the SNP markers were found to be biallelic, and none of the SNPs were tri-allelic since this is not detectable by KASP assays. Furthermore, none of the markers were read with null alleles and all the SNPs were validated as agreeing with the predicted nucleotide bases for their individual loci.

For quantitative characterization, several diversity measurements (Table 1) were calculated for each of the SNP markers based on the allele × genotype matrix generated by the KASP assays. In the first characterization step, the polymorphism information content (PIC) values for the markers was found to average 0.365 and range narrowly from 0.201 to 0.584 with the highest value being for AM24210 and the lowest value for AM20533. Values near 0.5 corresponded to the theoretical maximum PIC for biallelic markers according to Anderson (1993). The major allele frequency (MAF) averaged 0.676 and ranged from 0.388 to 0.871 with the same SNPs as highest and lowest as for PIC values, respectively. The gene diversity (GD) value of SNP loci averaged 0.439 and ranged from 0.225 to 0.654 with the highest and lowest values inverted for the two SNPs mentioned above. A total of four SNPs presented gene diversity values less than 0.3 and would not be recommended for subsequent work, whereas four SNPs had values higher than 0.6 and could be considered ideal in the future. The GD values were positively and significantly associated with PIC values (r = 0.982, *p* < 0.001) using Pearson Correlation coefficient. Additionally, this was correlated with lower MAF values (r = −0.972, *p* < 0.001). Correlation of PIC and MAF was also negative and significant (r = −0.944, *p* < 0.001).

### 3.2. Relationships between Grain Amaranth Accessions

**Figure 1:**
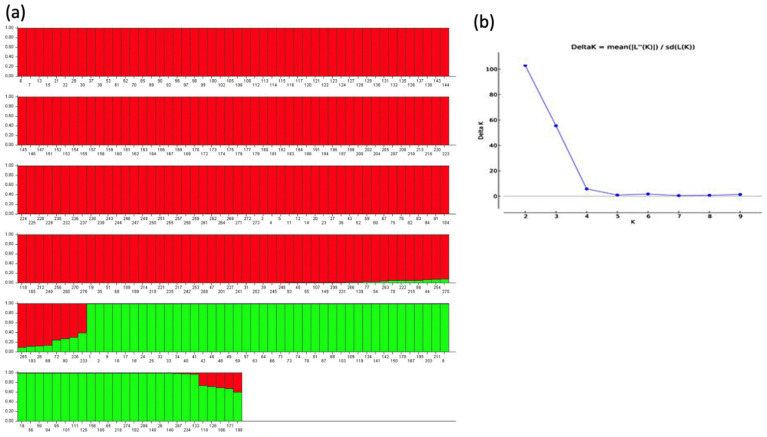
Population structure (**a**) and Evanno test (**b**) for K = 2 clusters and model-based ancestry of accessions with 200,000 burn-ins and 1,000,000 MCMC. Columns represent individual genotypes (numbered on *x*-axis) and genome admixture (*y*-axis) in populations structure. Abbreviations in Evanno test are for subpopulation number (K on the *x*-axis), and difference in probability for subpopulation (DeltaK on the *y*-axis). Red and green colors represent genomic representation for the different sub-populations according to best fit of the Evanno test. Both colors found in bars representing genotypes with admixture and the proportion of each subpopulations’ genetic contribution is shown by a crossing line dividing the two colors within the column.

Population structure analysis of the grain amaranth accessions revealed the highest ∆K value was found at K = 2 (Figure 1), indicating two primary populations in the collection. These were made up of South American accessions of the species *A. caudatus* along with *A. quitensis* and the Central and North American accessions of either *A. cruentus* or *A. hypochondriacus*. The detailed study of the Q bar plot for all the genotypes (Supplementary Materials, Figure S1) showed that population I comprised 204 accessions, population II comprised 63 accessions and the admixture group comprised nine accessions. Among 276 accessions, population I had 119 accessions of *A. cruentus*, 10 accessions of *A. hybridus*, 43 accessions, of *A. hypochondriacus*, seven accessions of *A. powelli*, and 25 SSE genotypes. Population II had 33 accessions of *A. caudatus*, 13 accessions of *A. quitensis*, 11 accessions of *A. hybridus*, one accession of *A. hypochondriacus*, one accession of *A. cruentus* and four accessions of SSE. The admixture group was decided based on a threshold value of membership coefficient to neither group, i.e., Q of 0.85. This group of intermediates between the populations consisted of five accessions of *A. hybridus*, two accessions of *A. retroflexus*, and one accession of *A. australis.*

Color coding for the Q coefficient in the structure figure showed that most shared alleles were found between *A. cruentus* and *A. hypochondriacus*. Among the three species of grain amaranths, *A. cruentus* was found to be most diverse followed by *A. hypochondriacus* and finally *A. caudatus*. This was supported by the Sum of squares within the population (SSWP) from the AMOVA analysis, where SSWP was 1242.7 for *A. cruentus*, 491.0 for *A. hypochondriacus* and 261.8 for *A. caudatus.*

The analysis of molecular variance (AMOVA) revealed high genetic variability within and among species (Table 2), and higher variance among species, than among individuals within species or within individuals. The estimated fixation index or total inbreeding coefficient (F_IT_) of 0.759 represented intra-species level diversity.

The inbreeding coefficient value of individuals within species (F_IS_) was 0.498 and the proportion of total genetic variance among species (F_ST_) was 0.52 and fixation index among individuals within total population (F_IT_) was 0.75 and all the values were highly significant (p < 0.001). The pairwise F_ST_ estimation showed smallest values of 0.062 among *A. caudatus* and *A. quitensis*, 0.291 among *A. caudatus* and *A. hybridus* and highest value of 0.703 among *A. caudatus* and *A powellii*, 0.717 among *A. caudatus* and *A. retroflexus*, and 0.752 among *A. quitensis* and *A. retroflexus* (Table 3).

Regarding the F_ST_ relationship among populations of different origins (Supplementary Materials, Table S3) the smallest F_ST_ of 0.06, 0.081 and 0.157 were observed between South America and Europe, South America and North America and South America and Central America, respectively. The highest F_ST_ were 0.72 and 0.71 between Asia and Europe or Asia and North America, respectively.

### 3.3. Cluster Analysis

**Figure 2:**
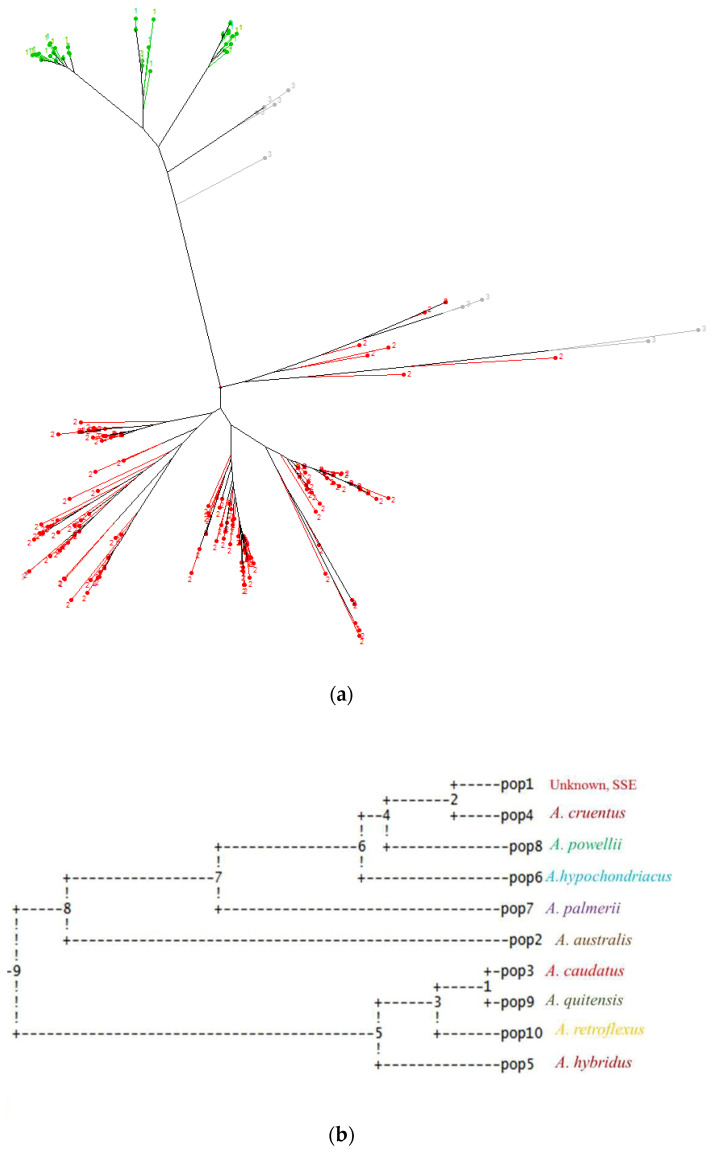
Relationships between accessions of Amaranths by (**a**) clustering with an UPGMA neighbor joining tree showing three major subgroups (subpopulation 1 shown in green colored dots, consisting of *A. caudatus* grain amaranths and *A. quitensis* wild relative accessions; subpopulation 2 in red colored dots consisting of *A. cruentus* and *A. hypochondriacus* grain amaranths; subpopulation 3 in gray dots and lines consisting of other species and weedy relatives) and (**b**) phylogenetic analysis of the USDA and SSE cultivars form nine species based on Nei [45] with numbers representing steps in species differentiation.

Relationship in the full set of accessions were visualized with a neighbor-joining (NJ) tree based on 44 polymorphic SNP markers (Figure 2). The genetic relationship between two populations demonstrated by STRUCTURE was further supported by neighbor-joining method of DARwin software. Neighbor- joining method of DARwin revealed two distinct clusters. Cluster 1 demonstrated most accessions from Central America followed by Asia, Africa, Europe and North America. Cluster 2 had most accessions from South America and North America. Cluster 1 was mostly represented by accessions of *A. cruentus* and *A. hypochondriacus*; while Cluster 2 was mostly represented by accessions of *A. caudatus* followed by *A. quitensis* and *A. hybridus*.

Accessions collected from SSE were of unknown species. They were found to be clustered in cluster 1, which shows the close relationship between these accessions with *A. hyochondriacus* and *A. cruentus* rather than *A. caudatus*.

## 4. Discussion

Information about genetic diversity among and within crop species is important for effective utilization of plant genetic resources especially from core collections derived from germplasm banks [18]. Analyses of genetic diversity have direct benefits in research related to evolution and population structure [42]. Various morphological and molecular markers have been used for the study of genetic diversity and evolutionary relationship in selected species of the genus *Amaranthus*. In this study, we found KASP markers from Maughan et al. [26,27] to be useful for classifying grain amaranth species and differentiating them from or grouping them with weedy species. The selection of SNP loci was based on their physical location across 16 chromosomes of the *A. caudatus* genome [47].

In a first observation, we saw good cross amplification with KASP markers across species along with bi-allelic polymorphism with low levels of observed heterozygosity (H_o_). Among the 45 SNP markers, only AM19583 which was mentioned to be polymorphic by Maughan et al. [26] would not show amplification in any of our experimental species. The other 44 SNPs all showed cross-amplification between species and were found to be of good polymorphism value. Most of the markers (32 out of 44 loci) had low H_o_ between 0.000 and 0.15. 

This was to be expected as the USDA accessions were primarily multiplied and grown out as self-pollinating plants. Overall H_o_ averaged 0.1215. The markers with values above 0.6 were AM17977, AM19378, AM19426 and AM22649 with the highest of all values at 0.750. Relatively high heterozygosity was also observed for AM18185 and AM21336, which were above 0.3; and for AM17870, AM19559 and AM20533, which were above 0.2 but below 0.3 (Table 1).

Some heterozygosity could be expected since grain amaranths are monoecious and have a moderate rate of inter plant hybridizaton in the field; while all the weedy accessions are highly outcrossing in nature. The two dioecious wild/weed species, *A. australis* and *A. palmeri*, represented obligate outcrossing plant types. The diversity exhibited by these SNP markers suggest that they can be efficiently used in future molecular breeding, marker assisted selection or diversity studies of Amaranths germplasm. The diversity result obtained from this study is likely to be unbiased since the markers were chosen from all chromosomes and a large number of accessions were used from each species.

In a second important aspect of our results, we found the population structure for the grain amaranths and even some wild relatives. Principally, this was based on South American species (*A. caudatus* and *A. quitensis*) compared to North American species (*A. cruentus* and *A. hypochondriacus*). The UPGMA analysis of our data showed association between geographical origin and genetic similarity. Most of the accessions from the same species and close geographical origins were clustered together. Phylogenetic analysis showed that the species were divided based on these continental and sub-continental origins; however, the placement of weed relatives did not agree with the more accurate sequence-based results of genotyping by sequencing in Amaranths [34,35].

Thirdly, the population structure analysis conducted at high burn-in length of 200,000 and MCMC values of 1,000,000. identified K = 2 subpopulations in our study. These two sub-populations had a few admixture accessions in between them. The occurrence of admixtures indicated possible crossing and hybridization between different wild, weedy or cultivated species of amaranths. The first population was found to have more di verse genotypes than second population. This could have been due to the uneven number of genotypes and species in each grouping.

Fourthly, in terms of the phylogenetic relationship between *Amaranthus* spp., the output from the PopGen software revealed two different genetic clusters (Figure 2b). The first cluster was comprised of *A. cruentus* and *A. hypochondriacus* accessions primarily, as well as all the *A. powellii*, *A. australis*, and *A. palmerii* gentoypes, along with *A. australis* and US cultivated accessions from SSE. A second cluster included *A. caudatus*, *A. quitensis*, *A. retroflexus* and some of the *A. hybridus* sampled.

Inferences from these results are that the US cultivars are mainly from the North American grain amaranths, *A. cruentus* and *A. hypochondriacus*, which were closely related to each other and likely to hybridize inter-specifically. Meanwhile, for South American species, a high amount of admixture was found between *A. caudatus* and what can be called a wild-weedy species, *A. quitensis.*

The possible progenitor species, *A. hybridus*, would be important in the formation of this latter group based on the limited number of accessions of this weed species that we studied. The close distance between *A. cruentus*, *A. hypochondriacus* and *A. powelli* depicted by pairwise F_ST_ values (Table 2), dendrogram and phylogenetic relationships (Figure 2) inferred the latter’s status as a possible progenitor for this North American group.

The dendrogram showed a clear differentiation between North and South American accessions. The first clusters represented accessions from Central America and North America with a few from Africa, Asia and Europe. The second clusters represented accessions from South America of the species *A. caudatus*, *A. quitensis* and *A. hybridus.* The status of the weed species *A. retroflexus* and *A. palmeri*, as well as the wild outgroup species *A. australis*, also known as the tree amaranth a native of Florida swamps, were inconclusive and could have been limited by the number of markers used.

These molecular marker results differed from morphologically based classification of the same accessions, which tended to group unrelated genotypes together [38]. In that study, ten clusters were found, based on morphological descriptors/markers, but these did not align with species differences. However, our results showing low differentiation levels between South American species coincided with the findings of Hauptli and Jain [48], who saw a close relationship between *A. cau datus* and *A. quitensis* based on allozyme markers. Similarly, the relationship and genetic clustering we found for *A. cruentus* and *A. hypochondriacus* was consistent with findings of Chan and Shun [20]. Any variation in results obtained from different studies may be because of differences in marker systems used, variation in number of species/accessions sampled and discrepancies of methods of data analysis. Interestingly, we found in the percentage of polymorphic loci among different species, comparatively lower number of polymorphic loci in *A. caudatus*, which support the findings previous authors on [3,49] the presence of less genetic variation in that species.

Overall, our results give some clues to the origin of grain Amaranths based partly on their geographic distribution. Clustering was found for *A. caudatus* and *A. quitensis* from South America compared to. *hypochondriacus*, *A. powellii* and *A. cruentus* from North and Central America. This supports the hypothesis of Sauer (1967) that *A. caudatus* originated from *A. quitensis* in South America and *A. hypochondriacus* and *A. cruentus* originated from *A. powellii* in Mexico. It appears that limited inter-species gene flow has occurred between *A. caudatus* and the other two cultivated species. Saur [3] observed no F_1_ hybrids from these combinations and crossing barriers between South American and North American types. One wild species, *A. hybridus*, may have played a role in domestication on both continents due to its wide distribution [48]. However, more accessions of this weedy species and individuals of hybrid origin should be analyzed to confirm its role in domestications. New methods of emasculation [49] could be used to empirically evaluate crossing ability for cultivars and wild or weedy accessions and to recreate domestication processes; and high throughput sequencing [50] against a reference pan-genome for *Amaranthus* could prove the number of chromosomes and genome characteristics of such hybrids.

## 5. Conclusions

Overall, the SNP markers showed a high level of polymorphism. These SNP markers could be efficiently used in crop improvement of grain amaranths or basic genetic studies of *Amaranthus*, QTL mapping and molecular breeding would be examples of this. A higher level of genetic diversity was seen in the accessions from North America than in South America. The accessions collected from Asia, Africa and Europe seemed to be closer to Central and North American accessions than to South American accessions. In this research, we used a large number of genotypes *per* cultivated species to increase reliability of our comments about domestication and because these are of more interest from a crop improvement standpoint. However, future studies should collect more wild genotypes to make further inferences about domestication source populations for cultivars and also the finer scale phylogenetic relationships between species. These wild accessions gave clues as to the origins of grain amaranth species as well as current active hybridization between some species. For example, *A. caudatus* and *A. quitensis* were closely related and intermingled at the population structure level, indicating one as a derivative of the other as well as continued introgression perhaps as hybrid swarms and high outcrossing rate.

The close relationship of *A. cruentus* and *A. hypochondriacus* suggests they do not have crossing barriers, that they may have hybridized in the past, that they may be hybridizing in secondary centers of diversity like Africa and Asia and that the two species can be used together in future breeding work. Directed marker introgression using the SNPs we found to be most distinct among the accessions could be used to encourage selection amongst such hybrids between these two species. Furthermore, the accessions with high yield from one species could be selected for crossing with the other and further research on hybrid crop improvement conducted. Grain amaranths have a great future as a highly nutritious crop for various regions of the world, and it is time for the full genetic resources of this group of species to be utilized in breeding programs.

## Figures and Tables

**Table 1:** Genetic diversity for 44 SNP markers evaluated for 276 *Amaranthus* accessions.

SNP Name	SNP Type	MAF	GD	H	PIC
AM17977	C/T	0.5888	0.5208	0.4094	0.4283
AM18039	A/G	0.7156	0.4280	0.0109	0.3664
AM19011	C/T	0.6920	0.4348	0.0000	0.3520
AM19559	C/T	0.8533	0.2534	0.2717	0.2263
AM19643	G/T	0.6703	0.4949	0.1957	0.4433
AM19746	A/G	0.4891	0.5277	0.1812	0.4158
AM20177	A/G	0.8007	0.3268	0.1014	0.2858
AM21724	A/G	0.7772	0.3576	0.1196	0.3111
AM21859	A/T	0.4783	0.6347	0.0072	0.5631
AM22137	A/C	0.7373	0.3892	0.0036	0.3163
AM22892	A/G	0.8116	0.3072	0.1159	0.2622
AM23006	A/C	0.6775	0.4437	0.0145	0.3545
AM23128	C/T	0.7428	0.3875	0.0145	0.3204
AM23196	C/T	0.8188	0.3004	0.0290	0.2613
AM23262	G/T	0.5417	0.5891	0.0109	0.5159
AM24029	A/G	0.7373	0.3946	0.0036	0.3273
AM24210	C/T	0.3877	0.6581	0.0000	0.5837
AM24401	C/G	0.7391	0.3911	0.0072	0.3226
AM27616	A/G	0.7482	0.3820	0.0036	0.3169
AM27642	A/G	0.4275	0.6534	0.0000	0.5800
AM17870	A/G	0.6232	0.4826	0.2681	0.3824
AM18185	C/T	0.7591	0.3675	0.0326	0.3026
AM19378	G/T	0.5960	0.4873	0.6268	0.3754
AM19426	A/T	0.6178	0.4951	0.6993	0.4007
AM19707	C/G	0.6268	0.5285	0.0000	0.4649
AM19842	A/G	0.7301	0.4081	0.0616	0.3452
AM19855	C/T	0.4493	0.6213	0.0000	0.5419
AM20180	A/G	0.7138	0.4183	0.0072	0.3447
AM20403	C/T	0.4982	0.5337	0.0833	0.4249
AM20533	C/T	0.8714	0.2251	0.2500	0.2013
AM21310	A/T	0.7681	0.3562	0.0000	0.2928
AM21336	C/G	0.8406	0.2691	0.3043	0.2348
AM21842	A/G	0.5942	0.5286	0.1377	0.4440
AM22476	A/G	0.7409	0.3910	0.0036	0.3251
AM22487	C/G	0.6757	0.4472	0.2210	0.3594
AM22649	C/T	0.6141	0.4821	0.7500	0.3759
AM23703	A/G	0.5688	0.4997	0.0072	0.3852
AM24078	A/G	0.7917	0.3328	0.0036	0.2820
AM24266	C/G	0.8478	0.2591	0.0072	0.2274
AM24531	C/T	0.6884	0.4472	0.0580	0.3723
AM24819	A/G	0.6558	0.4718	0.2609	0.3871
AM26171	C/T	0.6993	0.4308	0.0072	0.3523
AM27610	A/T	0.7120	0.4162	0.0109	0.3382
AM27626	A/C	0.6975	0.4242	0.0471	0.3372
Mean		0.6776	0.4363	0.1215	0.3648

Abbreviations: MAF, major allele frequency; GD, genetic diversity; H, heterozygosity; PIC, polymorphism information content.

**Table 2:** Analysis of molecular variance of grain amaranth accessions based on population.

Source of Variation	Df	SS	MS	%	Type	Fixation Index	*p*-Value
Among species	6	2095.326	349.221	52%	F_ST_	0.520	0.001
Among individuals within species	240	1967.552	8.198	24%	F_IS_	0.498	0.001
Within individuals	247	678.000	2.745	24%	F_IT_	0.759	0.001
Total	493	4740.879		100%			

Abbreviations: Df, degree of freedom; F_IT_, fixation index within individual; F_IS_, fixation index among populations; F_ST_, fixation index among individual within populations.

**Table 3:** Pairwise F_ST_ values among species of grain amaranths and wild relatives of the *Amaranthus* genus.

Species Name	*A. caudatus*	*A. cruentus*	*A. hybridus*	*A. hypo.*	*A. powellii*	*A. quitensis*	*A. retroflexus*
*A. caudatus*	0.000						
*A. cruentus*	0.643	0.000					
*A. hybridus*	0.291	0.345	0.000				
*A. hypochondriacus*	0.703	0.410	0.398	0.000			
*A. powellii*	0.703	0.377	0.334	0.424	0.000		
*A. quitensis*	0.062	0.635	0.306	0.702	0.714	0.000	
*A. retroflexus*	0.717	0.510	0.307	0.539	0.346	0.752	0.000

## Data Availability

Not applicable.

## Supplementary Materials

The following are available online at https://www.mdpi.com/article/10.3390/genes12121849/s1, Table S1: Passport data of Amaranthus accessions from the United States Department of Agriculture (USDA) used in this study, Table S2: Passport data of Amaranthus accessions from Seed Savers’ Exchange (SSE) used in this study, Table S3: Pairwise FST values among groups of Amaranthus accessions based on different geographical origins.
