# Supplementary material for: Relationship of Cultivated Grain Amaranth Species and Wild Relative Accessions
Source: Genes (Basel). 2021 Nov 23;12(12):1849. doi: 10.3390/genes12121849 (PMC8702087; doi:10.3390/genes12121849)
Supplement: Supplementary file 1 [file genes-12-01849-s001.zip › supplementary/Supplemental Table S1 final.pdf]

Table S1. Passport data of *Amaranthus* accessions from United States Department of Agriculture (USDA) used in this study.

Entry No	Entry type	Accession No.	Species	Origin Country	Origin Location	Name or Code
1	PI	553076	*A. australis*	United States	Florida	Giant Amaranth
2	PI	166045	*A. caudatus*	India	n.a.	Chua
3	PI	175039	*A. caudatus*	India	n.a.	RRC 10
4	PI	490440	*A. caudatus*	Peru	n.a.	LSK 19
5	PI	490477	*A. caudatus*	Peru	n.a.	LSK 58
6	PI	490491	*A. caudatus*	Argentina	n.a.	LSK 74
7	PI	490579	*A. caudatus*	Bolivia	n.a.	LSK 355
8	PI	490603	*A. caudatus*	Peru,	Cajamarca	LSK 398
9	PI	490604	*A. caudatus*	Bolivia,	Chuquisaca	HH 50
10	PI	490607	*A. caudatus*	Bolivia,	Chuquisaca	HH 54
11	PI	490609	*A. caudatus*	Equador,	Azuay	HH 66
12	PI	511679	*A. caudatus*	Argentina	n.a.	RRC 551
13	PI	511681	*A. caudatus*	Bolivia	n.a.	Coimi
14	PI	511685	*A. caudatus*	Peru	n.a.	HH 19
15	PI	511687	*A. caudatus*	Peru	n.a.	HH 21
16	PI	511688	*A. caudatus*	Peru	n.a.	Achita
17	PI	511689	*A. caudatus*	Peru	n.a.	Achita
18	PI	511691	*A. caudatus*	Peru	n.a.	Achita
19	PI	511693	*A. caudatus*	Peru	n.a.	Achita
20	PI	511694	*A. caudatus*	Peru	n.a.	RRC 572
21	PI	511698	*A. caudatus*	Peru	n.a.	HH 37
22	PI	511700	*A. caudatus*	Peru	n.a.	Achis jonjoli
23	PI	511701	*A. caudatus*	Peru	n.a.	Achis jonjoli
24	PI	511703	*A. caudatus*	Peru	n.a.	Coyo
25	PI	511704	*A. caudatus*	Peru,	Cajamarca	Coyo
26	PI	553073	*A. caudatus*	United States	New Jersey	Love-lies bleeding
27	PI	568132	*A. caudatus*	Bolivia,	Tarija	coime
28	PI	568147	*A. caudatus*	Bolivia,	Tarija	coime
29	PI	608019	*A. caudatus*	Ecuador,	Pichincha	HH75
30	PI	634914	*A. caudatus*	Pakistan,	Kashmir	RRC 1395
31	PI	642736	*A. caudatus*	United States	California	RRC 379
32	PI	642741	*A. caudatus*	Bolivia	n.a.	Cscar Blanco
33	PI	649224	*A. caudatus*	Peru,	Ancash	RRC899
34	PI	669838	*A. caudatus*	Bolivia	n.a.	RRC 556
35	Ames	30932	*A. caudatus*	Russia	n.a.	Red Utopia
36	Ames	31991	*A. cruentus*	United States	California	Burgundy Amaranth
37	PI	288278	*A. cruentus*	India	n.a.	RRS 31
38	PI	433228	*A. cruentus*	Guatemala	Chimaltenan go	2
39	PI	451711	*A. cruentus*	Mexico	n.a.	GN 1144
40	PI	451825	*A. cruentus*	Guatemala,	Chimaltenan go	Cr 045

41	PI	462371	*A. cruentus*	Sudan	n.a.	CN110
42	PI	477912	*A. cruentus*	Mexico	n.a.	RRC 416
43	PI	477913	*A. cruentus*	Mexico	n.a.	RRC 1011
44	PI	477914	*A. cruentus*	Mexico	n.a.	RRC 1041
45	PI	482049	*A. cruentus*	Zimbabwe	n.a.	TGR 542
46	PI	482051	*A. cruentus*	Zimbabwe	n.a.	TGR 603
47	PI	490655	*A. cruentus*	Peru	n.a.	RRC 1121
48	PI	490662	*A. cruentus*	Benin	n.a.	RRC 1034
49	PI	494771	*A. cruentus*	Zambia	n.a.	ZFA 3330
50	PI	494777	*A. cruentus*	Zambia	n.a.	ZFA 3653
51	PI	500264	*A. cruentus*	Zambia	n.a.	ZM 2255
52	PI	500267	*A. cruentus*	Zambia	n.a.	ZM 2309
53	PI	511715	*A. cruentus*	Guatemala	RRC	618
54	PI	511717	*A. cruentus*	Guatemala	HH	85
55	PI	511718	*A. cruentus*	Guatemala	HH	86
56	PI	511719	*A. cruentus*	Guatemala	n.a.	Niqua, alegria
57	PI	511726	*A. cruentus*	Mexico	n.a.	HH 99
58	PI	511727	*A. cruentus*	Mexico	n.a.	HH 100
59	PI	511732	*A. cruentus*	Guatemala	n.a.	HH 106
60	PI	511876	*A. cruentus*	Mexico	n.a.	Huatle
61	PI	515959	*A. cruentus*	U.S.	Montana	Montana-3
62	PI	527567	*A. cruentus*	Burundi	n.a.	IZ 32
63	PI	527570	*A. cruentus*	Rwanda	n.a.	IZ 166
64	PI	536437	*A. cruentus*	Maldives	n.a.	KLM 1863
65	PI	538320	*A. cruentus*	U.S.	Pennsylvania	K 283
66	PI	538321	*A. cruentus*	U.S.	Pennsylvania	K 436
67	PI	566896	*A. cruentus*	United States	Arizona	RRC 537
68	PI	566897	*A. cruentus*	India,	Kerala	Kerala Red
69	PI	566898	*A. cruentus*	Mexico	n.a.	RRC 362
70	PI	576447	*A. cruentus*	Nigeria,	Oyo	Unidentified
71	PI	576451	*A. cruentus*	Nigeria,	Oyo	ED82/1029A
72	PI	576456	*A. cruentus*	Nigeria,	Oyo	NHA/9A
73	PI	576457	*A. cruentus*	Nigeria,	Oyo	NHA/13A/
74	PI	576462	*A. cruentus*	Nigeria	n.a.	TB 81/790B
75	PI	576466	*A. cruentus*	Nigeria,	Oyo	NHA/30A
76	PI	576477	*A. cruentus*	Nigeria	n.a.	TE 81/27A
77	PI	578280	*A. cruentus*	Nigeria,	Oyo	NHA/15
78	PI	590992	*A. cruentus*	China	Tibet	n.a.
79	PI	604555	*A. cruentus*	Mexico,	Morelos	Mapes 818
80	PI	604556	*A. cruentus*	Mexico,	Morelos	Mapes 819
81	PI	604666	*A. cruentus*	United States	Pennsylvania	RRC 1027
82	PI	605353	*A. cruentus*	China	RRC	5445
83	PI	605354	*A. cruentus*	United States	Pennsylvania	K 112
84	PI	606767	*A. cruentus*	United States	Pennsylvania	K 277
85	PI	606798	*A. cruentus*	Mexico,	Mexico	RRC 1032
86	PI	607532	*A. cruentus*	Netherlands,	South Holland	Split Personality
87	PI	612170	*A. cruentus*	China,	Hubei	V67
88	PI	618962	*A. cruentus*	Benin	n.a.	RRC 67

89	PI	628780	*A. cruentus*	Mexico,	Morelos	RRC 423
90	PI	628781	*A. cruentus*	Mexico,	Morelos	RRC 444
91	PI	628782	*A. cruentus*	Mexico,	Morelos	RRC 446
92	PI	628783	*A. cruentus*	Mexico,	Morelos	RRC 776
93	PI	628784	*A. cruentus*	Mexico,	Puebla	RRC 1139
94	PI	628785	*A. cruentus*	Mexico,	Puebla	RRC1140
95	PI	628793	*A. cruentus*	Zaire,	Shaba	RRC 685
96	PI	633584	*A. cruentus*	China,	Beijing	RRC 27
97	PI	633585	*A. cruentus*	Guatemala,	Chimaltenan go	RRC 624
98	PI	633592	*A. cruentus*	Mexica,	Colima	RRC 1191
99	PI	641043	*A. cruentus*	Nigeria,	Oyo	CEN/IB/97/AMA0 03
100	PI	641045	*A. cruentus*	Nigeria,	Oyo	CEN/IB/97/AMA0 05
101	PI	642734	*A. cruentus*	Mexico	n.a.	RRC 1012
102	PI	642742	*A. cruentus*	United States	Pennsylvania	K 459
103	PI	643037	*A. cruentus*	Mexico,	Federal district	RRC 413
104	PI	643039	*A. cruentus*	Mexico,	Morelos	RRC 415
105	PI	673040	*A. cruentus*	Mexico,	Morelos	RRC 417
106	PI	643042	*A. cruentus*	Mexico,	Morelos	RRC 419
107	PI	643043	*A. cruentus*	Mexico,	Morelos	RRC 420
108	PI	643044	*A. cruentus*	Mexico,	Morelos	RRC 421
109	PI	643045	*A. cruentus*	Mexico,	Morelos	RRC 422
110	PI	643046	*A. cruentus*	Mexico,	Morelos	RRC 424
111	PI	643047	*A. cruentus*	Mexico,	Morelos	RRC 425
112	PI	643048	*A. cruentus*	Mexico,	Morelos	RRC 426
113	PI	643049	*A. cruentus*	Mexico,	Morelos	RRC 428
114	PI	643051	*A. cruentus*	Mexico,	Morelos	RRC 430
115	PI	643052	*A. cruentus*	Mexico,	Morelos	RRC 431
116	PI	643053	*A. cruentus*	Mexico,	Morelos	RRC 432
117	PI	643054	*A. cruentus*	Mexico,	Morelos	RRC 433
118	PI	643055	*A. cruentus*	Mexico,	Morelos	RRC 434
119	PI	643056	*A. cruentus*	Mexico,	Morelos	RRC 435
120	PI	643057	*A. cruentus*	Mexico,	Morelos	RRC 436
121	PI	643060	*A. cruentus*	Mexico,	Morelos	RRC 441
122	PI	643062	*A. cruentus*	Mexico,	Morelos	RRC 443
123	PI	643064	*A. cruentus*	Mexico,	Morelos	RRC 447
124	PI	643077	*A. cruentus*	Mexico,	Morelos	RRC 463
125	PI	643078	*A. cruentus*	Mexico,	Oxaca	RRC 464
126	PI	647848	*A. cruentus*	United States	California	RRC 548
127	PI	649506	*A. cruentus*	Mexico,	Tlaxcala	RRC 483
128	PI	649408	*A. cruentus*	Mexico,	Tlaxcala	RRC 491
129	PI	649511	*A. cruentus*	Mexico,	Tlaxcala	RRC 499
130	PI	649512	*A. cruentus*	Mexico,	Tlaxcala	RRC 500
131	PI	649513	*A. cruentus*	Mexico,	Tlaxcala	RRC 501
132	PI	649514	*A. cruentus*	Mexico,	Tlaxcala	RRC 506
133	PI	649515	*A. cruentus*	Mexico,	Puebla	RRC 733

134	PI	649516	*A. cruentus*	Mexico,	Puebla	RRC 734
135	PI	649517	*A. cruentus*	Mexico,	Puebla	RRC 735
136	PI	649519	*A. cruentus*	Mexico,	Puebla	RRC 737
137	PI	649520	*A. cruentus*	Mexico,	Puebla	RRC 738
138	PI	649521	*A. cruentus*	Mexico,	Morelos	RRC 768
139	PI	649522	*A. cruentus*	Mexico,	Morelos	RRC 770
140	PI	649523	*A. cruentus*	Mexico,	Morelos	RRC 771
141	PI	649524	*A. cruentus*	Mexico,	Morelos	RRC 772
142	PI	649525	*A. cruentus*	Mexico,	Morelos	RRC 773
143	PI	649526	*A. cruentus*	Mexico,	Morelos	RRC 779
144	PI	649527	*A. cruentus*	Mexico,	Morelos	RRC 781
145	PI	649528	*A. cruentus*	Mexico,	Puebla	RRC782
146	PI	649603	*A. cruentus*	Mexico,	Puebla	RRC743
147	PI	649609	*A. cruentus*	Mexico,	Federal district	RRC 774
148	PI	649612	*A. cruentus*	Mexico,	Morelos	RRC 778
149	PI	658727	*A. cruentus*	Guatemala	n.a.	RRC 384
150	PI	658729	*A. cruentus*	Cen. Afr. Republic	n.a.	RRC 844
151	PI	658731	*A. cruentus*	United States	Iowa	Y-004-W
152	PI	664489	*A. cruentus*	Mexico,	Puebla	DB 2010884
153	PI	665285	*A. cruentus*	Venezuela,	Falcon	RRC 1265
154	PI	665286	*A. cruentus*	Venezuala	n.a.	RRC1266
155	PI	667160	*A. cruentus*	Guatemala	n.a.	RRC 626
156	PI	669937	*A. cruentus*	Tanzania	n.a.	RRC 25
157	PI	669938	*A. cruentus*	Tanzania	n.a.	RRC 56
158	PI	511684	*A. hybridus*	Peru	n.a.	Quihuicha
159	PI	511733	*A. hybridus*	Peru	n.a.	HH 48
160	PI	511734	*A. hybridus*	Bolivia,	Chuquisaca	HH 52
161	PI	511735	*A. hybridus*	Bolivia	n.a.	HH 53
162	PI	568179	*A. hybridus*	United States	Iowa	Ames 12991
163	PI	572255	*A. hybridus*	United States	California	Aberrant Amaranth
164	PI	608791	*A. hybridus*	United States	California	RRC 382
165	PI	669835	*A. hybridus*	Argentina	n.a.	RRC 550
166	PI	490489	*A. hybridus*	Peru	n.a.	RRC 1119
167	PI	490682	*A. hybridus*	Ecuador	n.a.	LSK 173
168	PI	490694	*A. hybridus*	Ecuador	n.a.	LSK 186
169	PI	500249	*A. hybridus*	Zambia	n.a.	ZM 1845
170	PI	511724	*A. hybridus*	Mexico	n.a.	HH 96
171	PI	603889	*A. hybridus*	United States	Ohio	Pop 42
172	PI	603895	*A. hybridus*	United States	Indiana	Pop 48
173	PI	604568	*A. hybridus*	Mexico,	Puebla	Mapes 831
174	PI	604602	*A. hybridus*	Mexico,	Puebla	Basurto 1614
175	PI	604673	*A. hybridus*	United States	Virginia	Ames 14358
176	PI	605351	*A. hybridus*	Greece	n.a.	RRC 847
177	PI	632247	*A. hybridus*	United States	North Carolina	DB 200128
178	PI	636178	*A. hybridus*	United States	Indiana	RRC 100B

179	PI	636180	*A. hybridus*	Colombia,	Cundinamarca	RRC 1153
180	PI	636181	*A. hybridus*	United States	Delaware	RRC 1195
181	PI	652416	*A. hybridus*	Brazil,	Goias	CPAC96-1
182	PI	667156	*A. hybridus*	Equador	n.a.	RRC 610
183	PI	667158	*A. hybridus*	Guatemala	n.a.	RRC 616
184	PI	669837	*A. hybridus*	Bolivia,	Tarija	RRC 553
185	Ames	5666	*A. hypochondriacus*	India	n.a.	RRC 1193
186	Ames	5667	*A. hypochondriacus*	India,	U.P	RRC 1194
187	PI	210995	*A. hypochondriacus*	Afganistan	n.a.	RRC 79
188	PI	274276	*A. hypochondriacus*	India,	Punjab	AVRDC 74-38
189	PI	274279	*A. hypochondriacus*	India,	H.P	RRC 171
190	PI	337611	*A. hypochondriacus*	Uganda	n.a.	P 373
191	PI	477915	*A. hypochondriacus*	India	n.a.	RRC 1008
192	PI	477916	*A. hypochondriacus*	Mexico	n.a.	RRC 1023
193	PI	477917	*A. hypochondriacus*	Mexico	n.a.	RRC 1024
194	PI	480592	*A. hypochondriacus*	India	n.a.	IC-38061-1
195	PI	480608	*A. hypochondriacus*	India	n.a.	IC-38087
196	PI	480711	*A. hypochondriacus*	India	n.a.	IC-38187
197	PI	480755	*A. hypochondriacus*	India	n.a.	IC-38221
198	PI	480918	*A. hypochondriacus*	India	n.a.	IC-38360
199	PI	481023	*A. hypochondriacus*	India	n.a.	IC-38527
200	PI	481226	*A. hypochondriacus*	India	n.a.	IC-42254-2
201	PI	481134	*A. hypochondriacus*	India	n.a.	IC-42255-5
202	PI	490752	*A. hypochondriacus*	Guatemala	n.a.	LSK 80
203	PI	490758	*A. hypochondriacus*	Mexico	n.a.	RRC 1023
204	PI	511721	*A. hypochondriacus*	Mexico	n.a.	HH 93
205	PI	511731	*A. hypochondriacus*	Mexico	n.a.	RRC 646

206	PI	511877	*A. hypochondriacus*	Mexico	n.a.	Huatle
207	PI	540446	*A. hypochondriacus*	Pakistan	n.a.	RRC 1004
208	PI	558499	*A. hypochondriacus*	United States	Nebraska	Plainsman
209	PI	568125	*A. hypochondriacus*	United States	Iowa	DB921
210	PI	576486	*A. hypochondriacus*	United States	California	UC126
211	PI	584523	*A. hypochondriacus*	United States	Colorado	Ames 21897
212	PI	599682	*A. hypochondriacus*	Mexico,	Sonora	RRC 541
213	PI	604461	*A. hypochondriacus*	Germany	n.a.	Elephant Head
214	PI	604576	*A. hypochondriacus*	Mexico,	Puebla	Mapes 841
215	PI	604577	*A. hypochondriacus*	Mexico,	Puebla	Mapes 847
216	PI	604796	*A. hypochondriacus*	Unknown	n.a.	RRC 147
217	PI	607534	*A. hypochondriacus*	Netherlands,	North Holland	Pygmy Torch
218	PI	618585	*A. hypochondriacus*	India,	Kerala	Ames 23941
219	PI	619237	*A. hypochondriacus*	Nepal	n.a.	RRC 124
220	PI	619239	*A. hypochondriacus*	Nepal	n.a.	RRC 138
221	PI	619259	*A. hypochondriacus*	Nepal	n.a.	RRC 266
222	PI	619266	*A. hypochondriacus*	Nepal	n.a.	RRC 283
223	PI	633589	*A. hypochondriacus*	Mexica,	Chihuahua	GN 975
224	PI	643038	*A. hypochondriacus*	Mexico,	Federal district	RRC 414
225	PI	643041	*A. hypochondriacus*	Mexico,	Morelos	RRC 418
226	PI	643068	*A. hypochondriacus*	Mexico,	Mexico	RRC 452
227	PI	649305	*A. hypochondriacus*	China,	Heilongjiang	HLJ-037
228	PI	649540	*A. hypochondriacus*	Mexico,	Tlaxaca	RRC 510
229	PI	649579	*A. hypochondriacus*	Mexico	n.a.	RRC 755
230	PI	649607	*A. hypochondriacus*	Mexico,	Morelos	RRC 767

231	PI	652432	*A. hypochondriacus*	Brazil,	Federal district	CPAC96-18
232	PI	654430	*A. hypochondriacus*	Nepal	n.a.	SP 124
233	PI	666334	*A. hypochondriacus*	United States	Iowa	DB2011434
234	PI	669856	*A. hypochondriacus*	India,	U.P	UC125
235	PI	669887	*A. hypochondriacus*	India,	U.P	UC175
236	PI	633586	*A. palmeri*	Senegal,	Dakar	RRC 654
237	PI	572257	*A. powellii*	Rwanda	n.a.	AMA 89/79
238	PI	572261	*A. powellii*	Germany	n.a.	AMA 57/81
239	PI	538793	*A. powellii*	Russia	n.a.	AJCO73
240	PI	572259	*A. powellii*	Slovakia	n.a.	AMA 69/79
241	PI	572260	*A. powellii*	France	n.a.	AMA 74/82
242	PI	604671	*A. powellii*	United States	Washington	Ames14356
243	PI	490454	*A. quitensis*	Peru,	Apurimac	LSK 34
244	PI	490466	*A. quitensis*	Peru	n.a.	LSK 46
245	PI	490706	*A. quitensis*	Equador	n.a.	LSK 203
246	PI	490708	*A. quitensis*	Equador	n.a.	LSK 205
247	PI	511736	*A. quitensis*	Bolivia	n.a.	HH 55
248	PI	511743	*A. quitensis*	Ecuador	n.a.	HH 69
249	PI	511745	*A. quitensis*	Ecuador	n.a.	HH 71
250	PI	511747	*A. quitensis*	Ecuador	n.a.	HH 73
251	PI	511751	*A. quitensis*	Peru	n.a.	Yuyu colorado
252	PI	568154	*A. quitensis*	Bolivia,	Tarija	aroma
253	PI	649246	*A. quitensis*	Peru,	Ancash	RRC 928
254	PI	652419	*A. quitensis*	Brazil,	Goias	CPAC96-4
255	PI	652428	*A. quitensis*	Brazil,	Federal district	CPAC96-14
256	PI	669830	*A. quitensis*	Equador	n.a.	RRC 596
257	PI	669836	*A. quitensis*	Argentina,	Santa Fe	RRC 552
258	PI	669839	*A. quitensis*	Peru	n.a.	RRC 560
259	PI	636366	*A. retroflexus*	Unknown	n.a.	RRC 112C
260	PI	649310	*A. retroflexus*	Mongolia	n.a.	E94193
