# Supplementary material for: Relationship of Cultivated Grain Amaranth Species and Wild Relative Accessions
Source: Genes (Basel). 2021 Nov 23;12(12):1849. doi: 10.3390/genes12121849 (PMC8702087; doi:10.3390/genes12121849)
Supplement: Supplementary file 1 [file genes-12-01849-s001.zip › supplementary/Supplemental Table S2 final.pdf]

Table S2. Passport data of *Amaranthus* accessions from Seed Savers' Exchange (SSE).

S.No.	SSE	Plant name
1	SSE 1	R158
2	SSE 3	Mayo Indian
3	SSE 4	R1017
4	SSE 5	R1011
5	SSE 104	Kerala Red
6	SSE 108	Tunisian
7	SSE 112	Jamaican Calalu
8	SSE 115	Kahlula
9	SSE 119	Black Leaved
10	SSE 132	Kellerman
11	SSE 15	Oeshburg
12	SSE 22	Prince's Feather
13	SSE 24	Hopi Red Dye
14	SSE 29	Rodale 127
15	SSE 30	Rodale 124
16	SSE 31	Rodale 152
17	SSE 34	Rodale R125
18	SSE 35	Rodale R158
19	SSE 38	Rodale 156
20	SSE 39	*A. hypochondriacus,* unknown
21	SSE 6	San Martin
22	SSE 7	Golden Giant
23	SSE 79	M. Dreadicus
24	SSE 80	*A. dedos,* unknown
25	SSE 86	Black Seed
26	SSE 92	Roter Meier
27	SSE 93	Roland
