# Supplementary material for: Relationship of Cultivated Grain Amaranth Species and Wild Relative Accessions
Source: Genes (Basel). 2021 Nov 23;12(12):1849. doi: 10.3390/genes12121849 (PMC8702087; doi:10.3390/genes12121849)
Supplement: Supplementary file 1 [file genes-12-01849-s001.zip › supplementary/Supplemental Table S3 final.pdf]

Table S3. Pairwise F_{ST} values among groups of *Amaranthus* accessions of different geographical origin.

Region	Asia	South America	North America	Europe	Central America	Africa	unknown
**Asia**	0.000						
**South America**	0.647	0.000					
**North America**	0.710	0.081	0.000				
**Europe**	0.721	0.060	0.000	0.000			
**Central America**	0.483	0.157	0.159	0.136	0.000		
**Africa**	0.230	0.323	0.337	0.286	0.185	0.000	
**unknown**	0.705	0.446	0.576	0.516	0.280	0.188	0.000
